# Complex regulation and multiple developmental functions of *misfire*, the *Drosophila melanogaster *ferlin gene

**DOI:** 10.1186/1471-213X-7-21

**Published:** 2007-03-26

**Authors:** Michelle K Smith, Barbara T Wakimoto

**Affiliations:** 1 Department of Biology and Center for Developmental Biology, University of Washington, 24 Kincaid Hall, Box 351800, Seattle, WA 98195-1800, USA

## Abstract

**Background:**

Ferlins are membrane proteins with multiple C2 domains and proposed functions in Ca^2+ ^mediated membrane-membrane interactions in animals. *Caenorhabditis elegans *has two ferlin genes, one of which is required for sperm function. Mammals have several ferlin genes and mutations in the human *dysferlin *(*DYSF*) and *otoferlin *(*OTOF*) genes result in muscular dystrophy and hearing loss, respectively. *Drosophila melanogaster *has a single ferlin gene called *misfire *(*mfr*). A previous study showed that a *mfr *mutation caused male sterility because of defects in fertilization. Here we analyze the expression and structure of the *mfr *gene and the consequences of multiple mutations to better understand the developmental function of ferlins.

**Results:**

We show that *mfr *is expressed in the testis and ovaries of adult flies, has tissue-specific promoters, and expresses alternatively spliced transcripts that are predicted to encode distinct protein isoforms. Studies of 11 male sterile mutations indicate that a predicted Mfr testis isoform with five C2 domains and a transmembrane (TM) domain is required for sperm plasma membrane breakdown (PMBD) and completion of sperm activation during fertilization. We demonstrate that Mfr is not required for localization of Sneaky, another membrane protein necessary for PMBD. The *mfr *mutations vary in their effects in females, with a subset disrupting egg patterning and causing a maternal effect delay in early embryonic development. Locations of these mutations indicate that a short Mfr protein isoform carries out ferlin activities during oogenesis.

**Conclusion:**

The *mfr *gene exhibits complex transcriptional and post-transcriptional regulation and functions in three developmental processes: sperm activation, egg patterning, and early embryogenesis. These functions are in part due to the production of protein isoforms that vary in the number of C2 domains. These findings help establish *D. melanogaster *as model system for understanding ferlin function and dysfunction in animals, including humans.

## Background

Membrane-membrane interactions play key roles in animal physiology and development, and one protein family that has been implicated in these interactions is the ferlins. In mammals, there are four ferlin genes: *dysferlin *(*DYSF*), *myoferlin *(*MYOF*), *otoferlin *(*OTOF*), and *ferlin*-*1-like 4 *(*FER1L4*) [1]. In *Caenorhabditis elegans*, there are two: *fertilization defective-1 *(*fer-1*) and *fertilization defective like-1 *(*ferl-1*) [2]. *D. melanogaster *has one ferlin gene, *misfire *(*mfr*) [3]. Thus far, ferlins have been characterized by multiple C2 domains (between four and six) and a transmembrane (TM) domain near the C-terminus [1]. C2 domains are independently folded units of ~130 amino acids that form parallel β-sheets connected by surface loops. They are present in many proteins involved in signal transduction and membrane trafficking, such as protein kinase C, phospholipase, and synaptotagmin, and have the capacity to bind substrates that include Ca^2+^, phospholipids, inositol polyphosphates, and phosphotyrosines [4-6].

The specific roles and binding partners of the ferlin C2 domains, which are denoted C2A-C2F, are largely unknown. At least a subset are proposed to bind Ca^2+ ^since the activities of mammalian and *C. elegans *ferlins are Ca^2+^-mediated [1,2,7-10]. A study using an *in vitro *assay to measure changes in fluorescence emission spectrum after Ca^2+ ^binding, suggested that the C2D domain of mouse OTOF has Ca^2+ ^binding capacity [9]. In addition, *in vitro *assays have been used to document differences among the C2 domains in human DYSF and MYOF. Of the six C2 domains in these proteins, only C2A, which resides closest to the N-terminus, was found to bind phospholipid vesicles in a Ca^2+ ^dependent manner [7,10].

The functional roles of ferlins are revealed by the detrimental effects of gene disruptions. An important example occurs in the skeletal muscles of mice and humans with mutations in the *DYSF *gene [11,12]. Current data suggest that DYSF, which is localized to intracellular vesicles, acts to repair skeletal muscle membrane tears by permitting vesicle fusion and creating a plasma membrane patch [1,8]. In humans lacking normal DYSF, muscle membrane tears are inefficiently repaired, and, consequently, two forms of muscular dystrophy, Limb Girdle Muscular Dystrophy type 2B and Miyoshi Myopathy, can develop [11,12].

Other members of the ferlin protein family have also been implicated in membrane-membrane interactions. Mutations in mammalian *OTOF *cause a form of autosomal recessive deafness known as DFNB9 due to problems with synaptic vesicle exocytosis in inner ear hair cells [9,13,14]. Although MYOF has not yet been associated with a disease phenotype, it is localized to closely apposed membranes in a cell-culture model of muscle differentiation and myoblasts from *MYOF *null mice do not fuse efficiently [10]. Mutations in *C. elegans fer-1 *affect the fusion of Golgi-derived membranous organelles with spermatid plasma membrane [2,15]. As a result, the spermatids fail to mature into motile sperm, causing male sterility. The functions of mammalian FER1L4 and *C. elegans *FERL-1 are not yet known [1,2].

Ohsako et al. [16] previously reported that the *D. melanogaster mfr *gene is specifically required for male fertility and fertilization. This conclusion was based on a phenotypic analysis of only a single mutant allele. Here we characterized the *mfr *gene and showed that it has a complex transcriptional profile, with testis- and ovary-specific promoters and alternatively spliced transcripts that predict multiple isoforms. Through studies of 11 *mfr *mutations, we confirmed a role for *mfr *in sperm function and discovered roles in egg patterning and early embryogenesis. Together, the molecular and genetic studies permitted a correlation of ferlin structure with function and provided evidence that *mfr *carries out its multiple developmental roles using different protein isoforms.

## Results

Mutations in the *mfr *gene have been recovered in two independent screens for male sterile mutations [16,17]. Here, we describe 11 alleles that were recovered from the screen of Wakimoto et al., [17] as strong male sterile mutations. We classified these mutations as arresting sperm activation based on similarity to the phenotype induced by mutations in the *snky *gene [18]. Genetic mapping and complementation tests with *mfr*^1 ^[16] confirmed these mutant alleles are new *mfr *alleles. To understand effects on ferlin function, we molecularly characterized the *mfr *gene and the mutant alleles.

### The *mfr *gene exhibits complex transcriptional regulation

**Figure 1:**
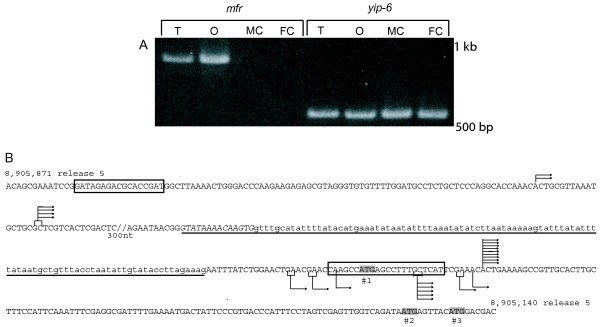
**The *mfr *gene is expressed in the testis and ovaries and uses multiple promoters**. In (A), an ethidium-bromide stained gel shows the results of an RT-PCR analysis of transcripts in testes (T), ovaries (O), male carcasses lacking reproductive organs (MC), and female carcasses lacking reproductive organs (FC). Gene specific primers for *mfr *or *yip-6*, the ubiquitously expressed ribosomal protein L5 gene [44], were used for each tissue sample. In (B), each arrow on the DNA sequence corresponds to a transcription start site represented by a 5' RACE product from testis (below the sequence) or ovary (above the sequence). Brackets indicate that either the G or A/C may serve as the actual transcription start site (see Methods). Also noted are sequences that are highly conserved among *Drosophila *species (boxed); potential start codons for the longest testis cDNA (T1) (gray ATG#1–3); and underlined intronic sequences in the 5' UTR of ovary transcripts (underlined lowercase letters are intronic in all six ovarian cDNAs, while underlined uppercase, italicized letters are also intronic in four of the cDNAs). Release 5.0 coordinate of the *D. melanogaster *genome sequence is indicated; 300 nts were omitted as indicated by the slashed lines.

As reported by Yamamoto and Ohsako [3], *mfr *corresponds to CG5747, whose structure was predicted by the *Drosophila *Genome Project [19]. The lack of *mfr *ESTs recovered from various cDNA screens [20,21] and failure to detect *mfr *transcripts on Northern blots containing poly A+ RNA from 1,000 testes, 500 ovaries, and male and female carcasses (data not shown), indicates that *mfr *transcripts may be rare. Consequently, we used RT-PCR to search for *mfr *transcripts and detected transcripts in the testes and ovaries, but not in the carcasses of adult flies that lacked gonads (Figure 1A).

To determine the *mfr *transcript structure, we performed 5' and 3' RACE using poly A+ RNA isolated from testes and ovaries. Sequence analysis of 10 testicular and 15 ovarian 5' RACE products revealed multiple transcriptional start sites (Figure 1B). All six of the identified testis transcription start sites mapped within a 35 bp region. This pattern of multiple nearby start sites is characteristic of a slippery promoter, which has previously been described by Yasuhara et al., [22] for other *Drosophila *genes. In contrast, the three identified ovarian transcription start sites mapped over a wider span of 516 bp, with each site distinct from those identified in the testis.

The *mfr *promoters lack the motifs identified by Ohler et al. [23] as common to *Drosophila *promoters. However, we note two regions of interest as putative regulatory sequences that are highly conserved among *Drosophila *species (Figure 1B). A 22 bp sequence, located just downstream of the 5' most testes transcription start site is also found in the homologous *mfr *region in *D. simulans, D. yakuba, D. ananassae*, and shows 86% identity in *D. pseudoobscura*. A 17 bp sequence which is located 87 bp upstream of the most 5' ovarian start site shows complete conservation in *D. simulans, D. yakuba, D. ananassae, D. pseudoobscura*, and 76% identity in the more distantly related *D. mojavensis*, and *D. virilis*. The location of this sequence is also within the 5' UTR of *Tsp66E*, which is transcribed from the opposite strand of *mfr *orthologs in all of these species. Therefore, it may be an important regulatory sequence for either gene.

Sequence of 12 testicular and 10 ovarian 3' RACE products indicated that *mfr *has a single site for polyadenylation, which is located 103 nt downstream of a predicted stop codon. Together, the 5' and 3' RACE studies defined a minimal gene size of 6.761 kb, extending from the most upstream ovarian start site to the polyadenylation site. An estimate of maximum gene size was provided by testing a transgene containing a 10.7 kb genomic fragment for its ability to rescue *mfr *mutant phenotypes. Two independent insertions of the transgene fully rescued the *mfr *male sterility in a single copy (Table 1) and also rescued a defect in egg patterning, which is described in more detail below, in two copies (Table 2). Compared to the 6.761 kb region, this transgene includes an additional 131 bp of upstream sequence and ~3.85 kb downstream of the polyadenylation site.

**Table 1:** Rescue of the *mfr *male sterile phenotype by transgenes.

Strain	Male Genotype^†^	n*	% Fertile Crosses	Average Progeny Yield
control (*mfr+*)	*w; P{w*^+*mc*^*mfr*^+*t*10.7^*}*A or B/+; *mfr*^*Z*0695^/*TM6*	10	100%	100+
control (*mfr-*)	*w*; +/*SM1*; *mfr*^*Z*0695^/*Df h-i22*	21	0%	0
transgenic (A)	*w; *+/*P{w*^+*mc*^*mfr*^+*t*10.7^*}*A; *mfr*^*Z*0695^/*Df h-i22*	11	100%	100+
transgenic (B)	*w; *+/*P{w*^+*mc*^*mfr*^+*t*10.7^*}*B; *mfr*^*Z*0695^/*Df h-i22*	25	100%	100+

^† ^A and B are two independent insertions of the transgene.

*number of single male crosses assayed

**Table 2:** Rescue of the *mfr *egg patterning defect phenotype by transgenes.

Strain	Female Genotype^†^	n*	% Eggs Patterning Defects
control (*mfr+*)	*w/w*^+^; *bw/SM1; st mfr*^*Z*1386^/*TM6 *or *w/w*^+^; *bw/SM1; st mfr*^*Z*0695^/*TM6*^∞^	293	9%
control (*mfr-*)	*w/w*^+^; *bw/SM1; st mfr*^*Z*1386^/*st mfr*^*Z*0695^	269	40%^Ω^
transgenic A	*w/w*^+^; *bw/P{w*^+*mc*^*mfr*^+*t*10.7^*}A;mfr*^*Z*1386^/*mfr*^*Z*0695^	197	21%^Ω^
transgenic B	*w/w*^+^; *bw/P{w*^+*mc*^*mfr*^+*t*10.7^*}B;mfr*^*Z*1386^/*mfr*^*Z*0695^	220	21%^Ω^
transgenic A two copies	*w; P{w*^+*mc*^*mfr*^+*t*10.7^*}A;mfr*^*Z*1386^/*mfr*^*Z*0695^	320	11%

^† ^A and B are two independent insertions of the transgene

*number of eggs assayed

^∞^siblings of the control (*mfr-*) females that have either the *mfr*^*Z*1386 ^or *mfr*^*Z*0695 ^mutation balanced over TM6

^Ω^percentage of defects is significantly different from control (*mfr*+) for control (*mfr-*)

P < 0.0001 R^2 ^= 0.05; for transgenic A, P = 0.0002 R^2 ^= 0.04; and for transgenic B, P = 0.0001 R^2 ^= 0.04.

### Differential splicing of *mfr *transcripts predicts tissue-specific protein isoforms

**Figure 2:**
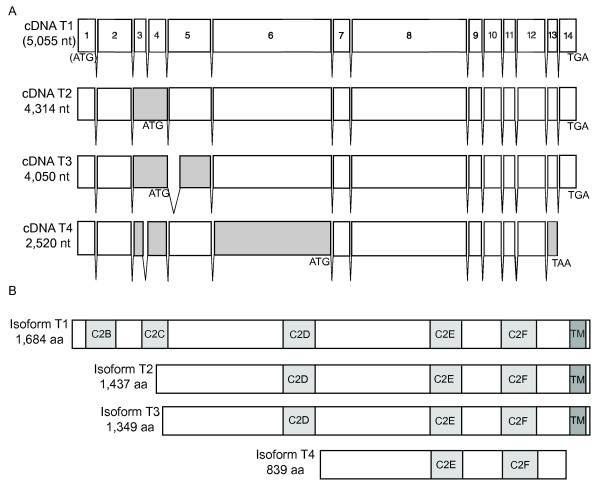
***mfr *testis cDNA analysis reveals alternatively spliced transcripts and predicts multiple isoforms**. For each cDNA shown in (A), the start and stop codons that define the longest ORFs and length of the ORF are indicated. For cDNA T1, the ATG and ORF length is deduced from additional 5'RACE and phylogenetic data as indicated by the parentheses. cDNA T1 is used as a reference to note where splicing differences give rise to alternative exons (gray). (B) shows the multiple Mfr protein isoforms predicted from testis cDNA analysis. The number of amino acids and positions of C2 (C2B-C2F) and transmembrane (TM) domains are noted for each predicted protein.

**Figure 3:**
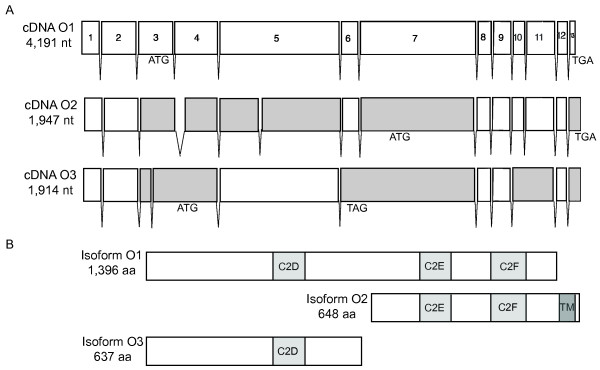
***mfr *ovarian cDNA analysis reveals alternatively spliced transcripts and predicts multiple isoforms**. For each cDNA shown in (A), the start and stop codons that define the longest ORFs and length of the ORF are indicated. cDNA O1 is used as a reference to note where splicing differences give rise to alternative exons (gray). (B) shows the multiple Mfr protein isoforms predicted from ovary cDNA analysis. The number of amino acids and positions of C2 and transmembrane (TM) domains are noted for each predicted protein.

We used the RACE results to design primer sets to recover near full-length *mfr *cDNAs from polyA+ selected testis and ovary RNA. Analysis of six testicular and nine ovarian cDNAs revised the CG5747 predicted gene structure [19] and identified differently spliced mRNAs with the potential to yield seven different Mfr protein isoforms (Figures 2 and 3).

The longest cDNA (T1), which was recovered from testis RNA, contains 14 exons and an open reading frame (ORF) with three in-frame Met codons located in exon 1 (Figures 1B and 2A). ATG#1 may be the initiating methionine codon for the mRNA corresponding to cDNA T1 because the sequences between ATG#1 and ATG#3 in *D. melanogaster *and its sister species are largely conserved (*D. simulans *with 93% identity, *D. sechellia *74% identity, *D. yakuba *74% identity, and *D. erecta *76% identity), suggesting a selective pressure to maintain the region as coding. The longest ORF predicts a protein with 1,684 amino acids, a TM domain near the C-terminus, and five C2 domains (Figure 2B). The C2 domains are denoted C2B-C2F based on their homology to C2 domains in mammalian ferlin proteins [2]. Three additional testis cDNAs (cDNA T2–T4) reflect alternative splicing events that increase the length of the 5' UTR and shorten the length of the ORF (Figure 2A). Corresponding protein isoforms are predicted to have two or three C2 domains, with the shortest isoform (T4) lacking the TM domain (Figure 2B).

Analysis of nine ovarian cDNAs revealed three different splicing variants, and all were different from those seen in testis cDNAs (Figure 3A). The ovarian transcripts vary in coding capacity and the length of the 5' UTR. Corresponding protein isoforms are predicted to have between one and three C2 domains, and only isoform O2 is predicted to have a TM domain (Figure 3B).

### *mfr *mutations indicate that male fertility requires the longest predicted Mfr isoform

For each of the 11 *mfr *mutations, we assayed the fertility of males that were homozygous or hemizygous in combination with deficiencies *Df(3L)h-i22 *or *Df(3L)ED4415*. We also tested the fertility of males with several *mfr *heteroallelic combinations (see methods). The result in all cases was strong recessive male sterility, with no progeny produced from any of the crosses between *mfr *males and wild-type females.

**Figure 4:**
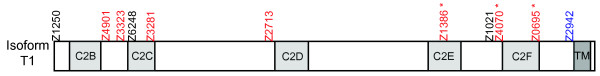
**Location of *mfr *mutations on testis protein isoform T1**. Locations of amino acid changes resulting from missense mutations are noted in black. Locations of protein truncations resulting from nonsense mutations are noted in red. The position of an altered exon predicted by a splice site mutation is noted in blue. Location of mutations that affect egg patterning are noted with a "*".

To understand how *mfr *mutation might affect ferlin function, we identified the molecular lesions in each of the 11 mutations. The mutations are distributed throughout the gene and provide information about the functional significance of predicted isoforms and specific residues (Table 3 and Figure 4). In particular, they implicate isoform T1 as essential for male fertility as it is the only isoform expected to be disrupted by all of the mutations. The missense mutation *mfr*^*Z*1250^, which changes a methionine to an isoleucine, provides additional evidence that ATG#1 (Figure 1B) encodes the initiating methionine. Also, *mfr*^*Z*6248^, which, like *mfr*^*Z*1250^, is expected to affect only isoform T1, changes a valine that is conserved in *D. simulans*, *D. sechellia*, *D. yakuba*, and *D. erecta *to aspartic acid in the region of the protein that is predicted to form the C2C domain. Because valine is a hydrophobic nonpolar amino acid and aspartic acid is a negatively charged amino acid, the *mfr*^*Z*6248 ^mutation likely disrupts C2C structure. In addition, *mfr*^*Z*1021 ^replaces a glycine, which is located between C2E and C2F, to a serine. This glycine is conserved in all known and predicted ferlin proteins in other species, indicating a key functional role in ferlins.

**Table 3:** Properties of the *mfr *mutations.

Allele	Mutation	Position on Chromosome^∞^	Amino Acid Change	Isoform T1 Amino Acid
*mfr*^*Z*1250^	G to A	8,905,275	M to I	1
*mfr*^*Z*4901^	G to A	8,904,738	W to *	156
*mfr*^*Z*3323^	C to T	8,904,596	Q to *	228
*mfr*^*Z*6248^	T to A	8,904,435	V to D	238
*mfr*^*Z*3281^	G to A	8,904,171	W to *	304
*mfr*^*Z*2713^	G to A	8,902,916	W to *	689
*mfr*^*Z*1386^	T to A	8,901,222	Y to *	1216
*mfr*^*Z*1021^	G to A	8,900,346	G to S	1396
*mfr*^*Z*4070^	G to A	8,900,329	W to *	1401
*mfr*^*Z*0695^	G to A	8,899,732	W to *	1522
*mfr*^*Z*2942^	G to A	8,899,358	Y to * (disrupts splice donor in intron)	1627

^∞ ^based on Release 5.0 of *D. melanogaster *genome sequence

Mapping of the mutations also revealed a disproportionately high number of nonsense mutations. Eight mutations are predicted to truncate isoform T1, either by mutating a splice junction (*mfr*^*Z*2942^) or in seven cases, by changing an amino acid to a stop codon (Table 3 and Figure 4). Three of the nonsense mutations, *mfr*^*Z*1386^, *mfr*^*Z*4070^, and *mfr*^*Z*0695 ^are the best candidates for *mfr *null mutations since they map to the C2E-C2F region that is shared by all of the predicted testis isoforms.

### *mfr *mutations consistently affect sperm function during fertilization

**Figure 5:**
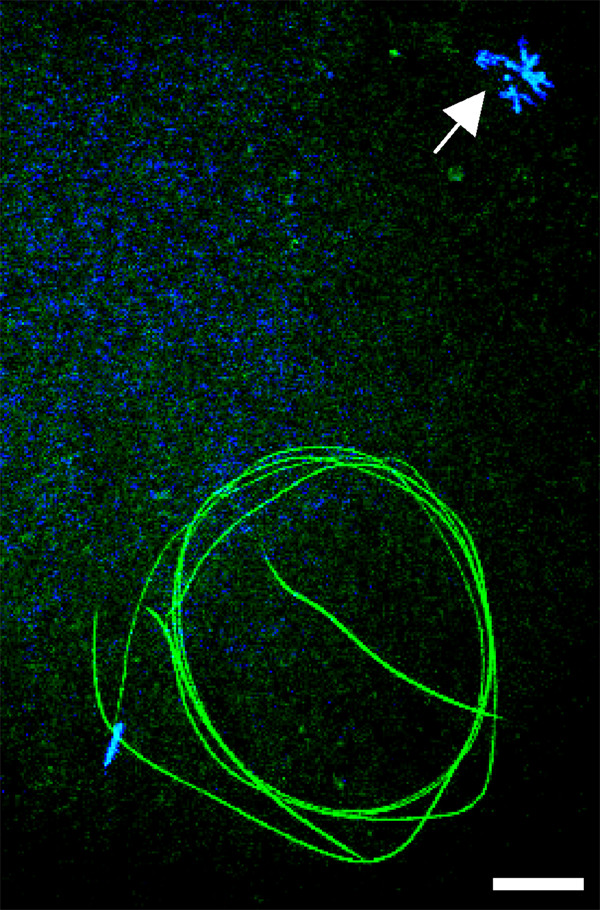
**The *mfr *sperm activation defect**. This confocal image shows an egg inseminated by sperm produced by a *dj-GFP;mfr*^*Z*0695^/*Df(3L)h-i22 *male. A DJ-GFP fusion protein (green) labels the sperm tail and reveals that entry into the egg, including the entire sperm tail, is complete. DAPI (blue) staining shows that the sperm nucleus remains condensed. The female meiotic products (arrow) also remain peripherally localized at the anterior end of the egg. Scale bar is approximately 20 microns.

To characterize the cause of *mfr *male sterility, we introduced a transgene that expresses the Don-Juan GFP (DJ-GFP) sperm tail marker into five different *mfr *mutant backgrounds, then monitored efficiency of sperm entry into the egg. Studies with a control strain show that the DJ-GFP assay detects 87% of the sperm entry events in eggs for up to 90 min after egg deposition (see methods). During normal fertilization, sperm entry is followed by events of sperm activation, including sperm plasma membrane breakdown (PMBD), nuclear decondensation, and formation of male pronucleus, in rapid succession. We found that in 85–100% eggs laid by wild type females mated to *mfr *males, sperm entered the egg (Table 4). However, nearly all retained a highly condensed nucleus (Figure 5). Three *mfr *alleles, including two nonsense alleles, occasionally allowed sperm to undergo nuclear decondensation and reach the pronuclear apposition stage, but not beyond (Table 4).

**Table 4:** Fertilization defects associated with *mfr *mutations

Male Genotype*	# of Eggs Examined	Percent Inseminated Eggs^∞^	Sperm Head Condensed^‡^	Subsequent Development^‡ ^(number of eggs)
*mfr*^*Z*0695^/*Df*	40	86%	100%	
*mfr*^*Z*6248^/*Df*	31	85%	100%	
*mfr*^*Z*2942^/*Df*	30	100%	93%	Sperm decondensation (1); Pronuclear apposition (1)
*mfr*^*Z*3281^/*Df*	30	92%	92%	Sperm decondensation (1); Pronuclear apposition (1)
*mfr*^*Z*2713^/*Df*	29	100%	90%	Pronuclear apposition (3)

* males express *dj-GFP *as sperm tail marker; *Df *is *Df(3L)h-i22*

^∞ ^percentage of inseminated eggs relative to a control cross of *dj-GFP *males mated to wild-type females; DJ-GFP was detected in 87% of the eggs

^‡ ^sperm chromatin visualized by DAPI staining

**Figure 6:**
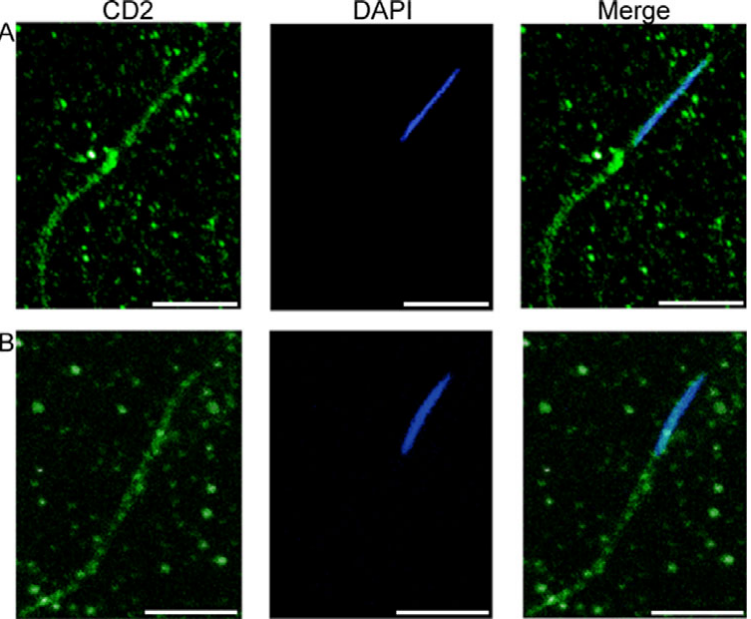
**Sperm produced by *mfr *males retain the CD2 membrane marker during fertilization**. For these confocal images, sperm produced by *mfr*^*Z*0695 ^males were assayed (A) in the testis or (B) after entry into the egg. CD2 (green) was detected by immunolocalization and nuclei (blue) were detected by DAPI staining. Merged images show that in the egg, CD2 persists around the head and tail, indicating failure in sperm PMBD. Scale bars are approximately ten microns.

Previous studies indicated that the *mfr*^1 ^allele [16], like *snky *mutations [18,24], affects PMBD. To monitor PMBD in eggs fertilized by sperm produced by *mfr*^*Z*0695 ^males, we used the method of Wilson et al. [24]. Specifically, the membrane protein CD2 was introduced into the sperm plasma membrane through expression of a CD2 transgene during spermatogenesis and its presence was monitored by immunolocalization before and after sperm entry into the egg. We found that the CD2 epitope was retained around the head and tail of *mfr*^*Z*0695 ^mutant sperm for at least 20 min after egg deposition in 83% of the eggs we monitored (n = 12, Figure 6), supporting an effect of *mfr *on PMBD.

**Figure 7:**
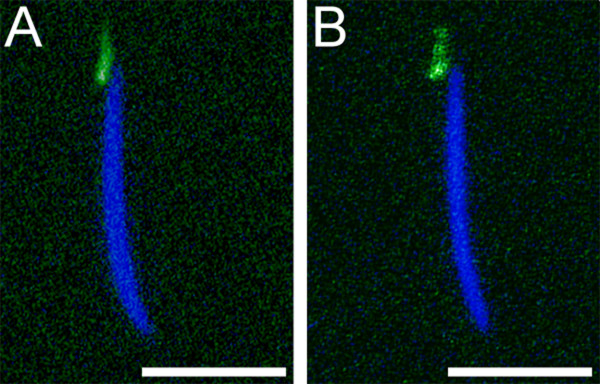
**Snky-GFP is localized properly in sperm produced by *mfr *males**. Confocal images show identical localization of Snky-GFP (green) to the acrosome of sperm produced by (A) wild-type and (B) *mfr*^*Z*0695 ^males. Nuclei (blue) were detected by DAPI-staining. Scale bars are approximately five microns.

Because *snky *is also important for sperm PMBD [18,24], Mfr could affect this event by controlling the localization of Snky. To test this idea, we introduced Snky-GFP into sperm produced by *mfr*^*Z*0695 ^males and assayed GFP expression. As shown in Figure 7, this *mfr *nonsense mutation does not alter the localization of Snky-GFP to the acrosomal membrane [24].

### A subset of *mfr *mutations affects egg patterning

**Figure 8:**
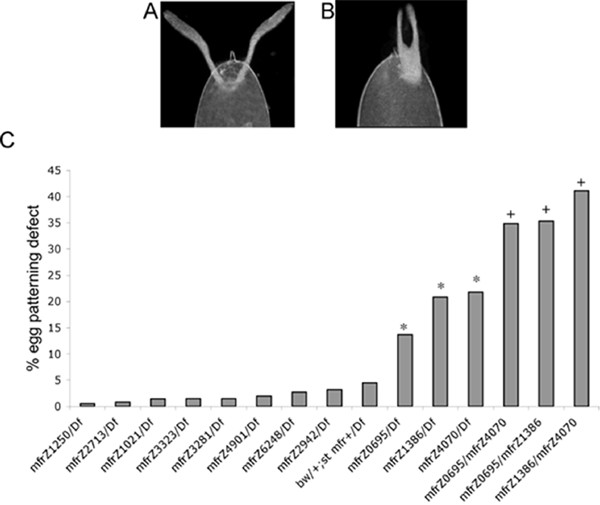
***mfr *mutations have differential effects on egg patterning**. Eggs produced by wild-type or *mfr *mutant females were monitored for (A) normal or (B) abnormal morphology of the dorsal appendages. Female genotypes are indicated with *Df *referring to *Df(3L)ED4415*. As shown in (C), three mutations, *mfr*^*Z*0695^, *mfr*^*Z*4070^, and *mfr*^*Z*1386^, induce significant effects on egg patterning. Asterisks (*) note a significantly higher percentage of defects compared to the *bw/+; st mfr*^+^/*Df *control (*P *< 0.0001, R^2 ^= 0.05 for *mfr*^*Z*0695^, R^2 ^= 0.07 for *mfr*^*Z*1386^, and R^2 ^= 0.10 for *mfr*^*Z*4070^). Plus signs (+) note a significantly higher percentage of defects associated with the heteroallelic condition compared to hemizygous condition for corresponding alleles (Table 5).

Expression of *mfr *transcripts in ovaries suggested a possible function during oogenesis. This role was confirmed by the discovery a recessive egg patterning phenotype induced by a subset of *mfr *mutations when expressed in females. The defect is visible in late stage eggs as abnormally short and closely apposed dorsal appendages (Figure 8A and 8B), a feature of the eggshell that signifies a ventralized egg chamber [25].

Penetrance of the egg patterning defect varied among the alleles (Figure 8C), with females hemizygous for the *mfr*^*Z*0695^, *mfr*^*Z*1386^, or *mfr*^*Z*4070 ^allele producing 14–22% abnormal eggs. The fact that these three nonsense alleles induced defects, but four nonsense mutations located upstream of the C2D encoding region do not (Figure 4), suggests that normal egg patterning requires a short Mfr isoform. Interestingly, two additional mutations that map downstream of C2D do not induce egg patterning defects. These mutations are *mfr*^*Z*1021^, which changes a glycine to a serine, and *mfr*^*Z*2942^, which mutates a splice junction to create a premature stop codon in some transcripts. Based on the locations of the nonsense mutations, the ovarian transcript that is important for egg patterning likely encodes the C2E and C2F domains, but does not use the splice junction affected by the *mfr*^*Z*2942 ^mutation. This structure is not represented among the three identified ovarian cDNAs.

We note that penetrance of the mutant phenotype in eggs produced by hemizygous females does not exceed 22% in any case, indicating that Mfr likely has a redundant function in ovaries. Surprisingly, we found that the percentage of egg patterning defects significantly increases to as high as 41% when the *mfr*^*Z*0695^, *mfr*^*Z*1386^, and *mfr*^*Z*4070 ^are placed in heteroallelic combinations (Table 5, Figure 8C). This observation verifies that Mfr proteins are expressed during oogenesis and suggests that the truncated Mfr proteins induce a dose-sensitive disruptive effect on egg patterning.

**Table 5:** χ^2 ^likelihood ratio tests comparing the percentage of egg patterning defects.

Female Genotype	*mfr*^*Z*0695^/*mfr*^*Z*4070^	*mfr*^*Z*0695^/*mfr*^*Z*1386^	*mfr*^*Z*1386^/*mfr*^*Z*4070^
*mfr*^*Z*0695^/*Df*	P < 0.0001 R^2 ^= 0.05	P < 0.0001 R^2 ^= 0.05	N/A
*mfr*^*Z*4070^/*Df*	P = 0.0053 R^2 ^= 0.01	N/A	P = 0.0002 R^2 ^= 0.02
*mfr*^*Z*1386^/*Df*	N/A	P < 0.0001 R^2 ^= 0.02	P < 0.0001 R^2 ^= 0.03

This χ^2 ^analysis shows that the percentage of egg patterning defects is significantly different when the eggs produced by *mfr*^*Z*0695^, *mfr*^*Z*4070^, and *mfr*^*Z*1386 ^over *Df (Df(3L)ED4415) *females are compared to females with these three alleles in heteroallelic combination.

**Figure 9:**
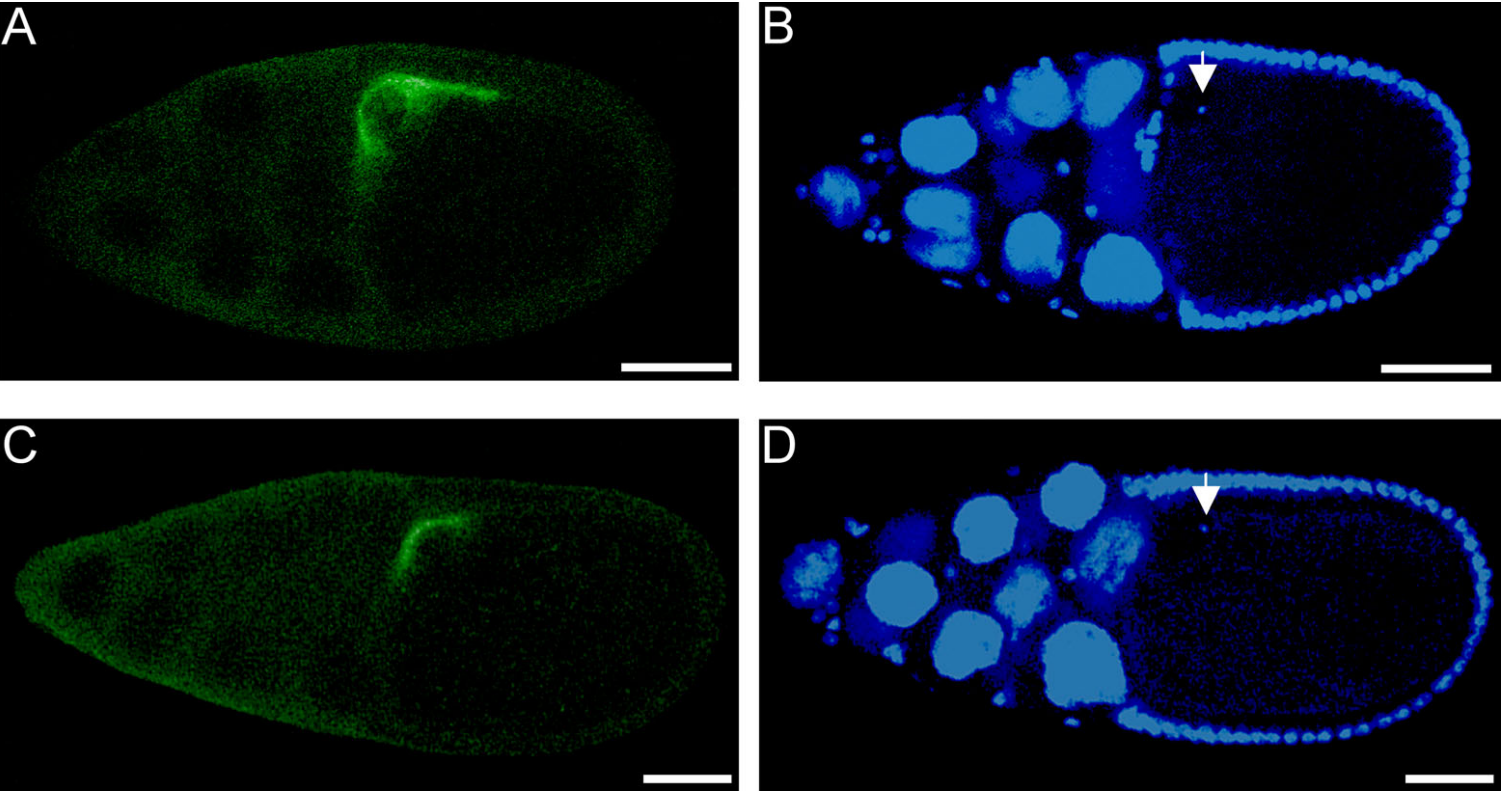
***mfr *mutations affect Gurken localization during oogenesis**. These confocal images compare a stage 10 egg chamber produced by a *bw;st *female (top panels) to one produced by a *mfr*^*Z*4070^/*mfr*^*Z*1386 ^female (bottom panels). Gurken (green) was detected by immunolocalization in dorsal-anterior corner of the wild-type oocyte (A) and in a more restricted region in the oocyte produced by *mfr *females (C). Nuclei were visualized by DAPI staining (blue) (B and D). Arrows indicate oocyte nucleus. Scale bars are approximately 50 microns.

Next, we investigated whether the defect might be accounted for by disruption of Gurken, a key ligand in the egg patterning pathway. In normal stage 10 egg chambers, Gurken shows a characteristic distribution in the anterior dorsal corner of the oocyte. This distribution permits proper signaling to overlying follicle cells [26]. Follicle cells that receive the Gurken ligand activate a downstream pathway that is responsible for the formation and proper spacing of the dorsal appendages [27]. Immunolocalization of Gurken revealed that in the majority of stage 10 egg chambers produced by control *bw;st *or *mfr*^*Z*4070^/*mfr*^*Z*1386 ^females, Gurken is properly localized (Figure 9A). However, in 15% of the *bw;st *egg chambers (n = 46) and 43% of the *mfr*^*Z*4070^/*mfr*^*Z*1386 ^egg chambers (n = 44), Gurken does not extend as far posteriorly (Figure 9B). A χ^2 ^likelihood ratio test reveals that this difference is statistically significant (*P *= 0.0025, R^2 ^= 0.0861).

### *mfr *mutations affect female fertility and the progression of embryogenesis

Mutations in *mfr *also affect embryonic development. Notably, only 43% of the total eggs laid by *mfr*^*Z*4070^/*mfr*^*Z*1386 ^mutant females mated to wild-type males hatch (n = 376 eggs). This hatch rate is similar for eggs with wild-type eggshell morphology and those with defective dorsal appendages; therefore, Mfr's role in embryogenesis may be separate from its role in egg patterning. Furthermore, the hatch rate of embryos of *mfr*^*Z*4070^/*mfr*^*Z*1386 ^mothers is significantly lower than the 84% hatch rate of embryos produced by the *mfr*^*Z*4070 ^or *mfr*^*Z*1386^/*TM6 *control strain (*P *< 0.0001 R^2 ^= 0.10 n = 340 eggs) and the 52% hatch rate observed for the *bw;st *parent strain (*P *= 0.0232 R^2 ^= 0.0049 n = 392 eggs).

**Figure 10:**
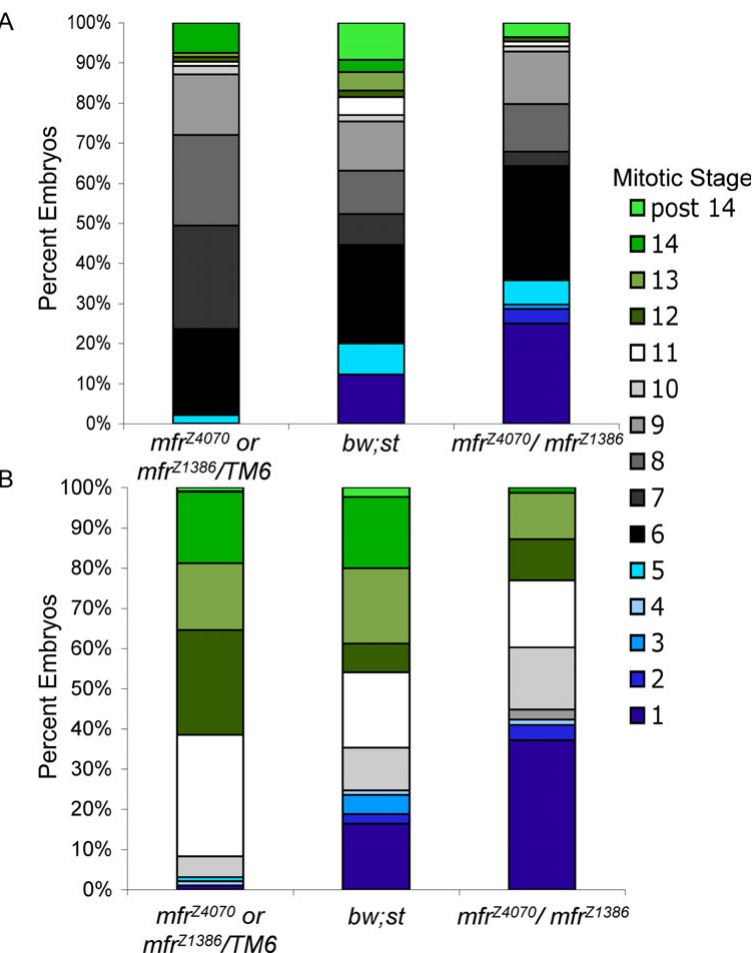
**Embryos produced by *mfr*^*Z*4070^/*mfr*^*Z*1386 ^mothers show developmental delay compared to embryos from *mfr+ *mothers**. Embryos were collected 50 min (A) and 90 min (B) after egg deposition, fixed, stained with DAPI and staged according to nuclear density.

To determine if the lower hatch rates are caused by visible defects during early embryonic development, *mfr*^*Z*4070^/*mfr*^*Z*1386 ^and control (*mfr*^*Z*4070 ^or *mfr*^*Z*1386^/*TM6 *and *bw;st*) females were mated to wild-type males and a minimum of 150 eggs were collected from each mating at 50 and 90 minutes after egg deposition. The embryos were fixed and stained with DAPI to visualize nuclei, and then classified according to mitotic cycle (Figure 10). This analysis revealed that the development of embryos produced from *mfr*^*Z*4070^/*mfr*^*Z*1386 ^mothers is delayed compared to embryos produced from mothers of the two control strains (*mfr*^*Z*4070 ^or *mfr*^*Z*1386^/*TM6 *and *bw;st*). Furthermore, an ordinal logistic regression analysis confirms that the effect of maternal strain on the stage of the embryonic development is statistically significant (P < 0.0001, X^2 ^= 58.31, d.f. = 2). This effect remains statistically significant when pairwise comparisons between the three strains are made.

Compared to *mfr*^*Z*4070 ^or *mfr*^*Z*1386^/*TM6*, embryos produced by *bw;st *females have low hatch rates. However, if the embryos arrested before a the end of cycle 1 are removed from the data set and a subsequent ordinal logistic analysis is performed, the effect of strain on mitotic stage is no longer significant between the *bw;st *parent strain and the *mfr*^*Z*4070 ^or *mfr*^*Z*1386^/*TM6 *control strain (*P *= 0.86, χ^2 ^= 0.03). This results suggests that embryos from *bw;st *mothers show defects in completing cycle 1, but if the embryos complete cycle 1, then the distribution of mitotic stages is similar to embryos from the *mfr*^*Z*4070 ^or *mfr*^*Z*1386^/TM6 mothers through cycle 14. Conversely, after the cycle 1 data is removed, maternal strain retains a significant effect on mitotic stage when embryos from *mfr*^*Z*4070^/*mfr*^*Z*1386 ^mothers are compared to embryos from both *bw;st *(*P *= 0.0016 χ^2 ^= 9.99) and *mfr*^*Z*4070 ^or *mfr*^*Z*1386^/TM6 (*P *< 0.0001 χ^2 ^= 15.40) mothers. This result indicates that mitotic defects in embryos from *mfr*^*Z*4070^/*mfr*^*Z*1386 ^mothers are not limited to the completion of cycle 1, but instead extend into later cycles.

## Discussion

We found that the *D. melanogaster *ferlin gene *misfire *is expressed in the testis and ovaries of adult flies, and has tissue-specific transcription initiation sites, alternatively spliced mRNAs, and multiple developmental functions. Our analysis of cDNAs, while not exhaustive, has provided a framework for predicting potential Mfr isoforms and for interpreting the differential effects of mutations on male and female fertility.

Thus far, ferlins have been described as having multiple C2 domains, typically four to six, and a TM domain at the C-terminus. Our cDNA study confirmed this ferlin structure for the predicted protein isoform T1, which we suggest from genetic data is the Mfr protein required for male fertility. However, we also isolated cDNAs that predict shorter testis and ovary isoforms. Alternative splicing appears to be common in the ferlin gene family [2,13,14,28-30]. For human *OTOF*, Northern blot analysis shows that both short and long transcripts are produced [13]. The functional significance of the short transcript is unknown, but its structure is strikingly similar to that predicted for Mfr isoforms T2 and T3. Also, a subset of *mfr *cDNAs (T4, O1, and O3) predicts isoforms that contain one to three C2 domains, but lack the TM domain. cDNAs that predict ferlin isoforms lacking TM domains have also been reported for human *MYOF *and *C. elegans fer-1 *[2,30]. Further biochemical studies are needed to determine which ferlin isoforms are produced and whether variation in C2 domains or presence of soluble versus membrane forms contributes to function.

Ferlin proteins are known to have a role in Ca^2+^-dependent membrane-membrane interactions in mammalian muscle cells, myoblasts, and inner ear hair cells [7-10], and *C. elegans *spermatids [2,15]. Therefore, Mfr may also mediate Ca^2+^-dependent interactions between membranes. In males, Mfr's time of action may be during spermatogenesis to affect sperm function during fertilization. However, its multiple effects on PMBD and later stages of sperm activation suggest activity of the protein during fertilization. After insemination, Mfr could facilitate interactions between the acrosome and sperm plasma membrane to elicit PMBD. These interactions may also involve Snky, an acrosomal membrane protein that like Mfr, we propose is acting as a signaling protein for PMBD [24]. Previously it was proposed that PMBD might occasionally occur spontaneously, allowing sperm produced by *snky *mutant males to effectively bypass the requirement for Snky and produce a few progeny [18]. Here, we show that a subset of sperm lacking Mfr function progress past PMBD to achieve nuclear decondensation and pronuclear apposition, but produce no progeny. Later events of sperm function, perhaps those associated with nuclear envelope dynamics or pronuclear apposition, may also require one or more Mfr isoforms. Possible Ca^2+ ^cues for Mfr activation may come from an intracellular source such as the acrosome, which is a Ca^2+^-storage vesicle in sperm of some species [31,32], or from cytoplasmic sources in the activated *D. melanogaster *egg [33].

Only three of the male sterile mutations, which are all located downstream of the C2D encoding region, induce detectable effects on female fertility. The locations of these mutations are consistent with the idea that a short ovarian isoform, which includes the C2E and C2F domains, is important for Gurken localization and consequently egg patterning. However, the incomplete penetrance of the *mfr *phenotype even with presumed null alleles *mfr*^*Z*0695^, *mfr*^*Z*1386^, and *mfr*^*Z*4070 ^suggests that other proteins can at least partially compensate for loss of Mfr. In addition, embryos produced by *mfr*^*Z*4070^/*mfr*^*Z*1386 ^mothers show developmental delay and/or arrest during the earliest mitotic divisions. During these early mitotic divisions, *D. melanogaster *embryos are rapidly dividing in a syncytium and do not undergo transcription. Consequently, the early mitotic cycles are under maternal genetic control. The defects observed in embryos produced from *mfr*^*Z*4070^/*mfr*^*Z*1386 ^mothers before cycle 13 suggest that *mfr *mRNA and/or proteins are maternally deposited into the embryo and used during the early mitotic divisions. Microdomains of Ca^2+ ^have been identified in *D. melanogaster *syncytial embryos undergoing mitosis [34] and Mfr may associate with these transient Ca^2+ ^signals that are critical for cell division.

For ferlin proteins, the contribution of individual C2 domains to ferlin function remains largely an unanswered question. In addition to biochemical analyses to address this question [7,9,10], these studies and those of Washington *et al*. using *C. elegans *[2] show that genetic analysis provides a complimentary approach. For the *C. elegans fer-1 *gene, a predominance (8/10) of missense mutations were recovered. The location of these mutations within the C2C-, C2E-, and C2F-encoding domains suggested that each of these domains is important for *fer-1 *function, and that there is little functional redundancy among the C2 domains. In contrast, we recovered an unusually high proportion of *mfr *nonsense mutations (8/11), which may reflect functional redundancy among C2 domains within the Mfr protein. Alternatively, there may be functional redundancy among protein isoforms expressed in the same tissue, with the introduction of premature stop codons expected to have more general effects on isoform production. However, one critical difference between the *C. elegans *and *D. melanogaster *studies is that the majority of the *C. elegans *mutations were selected as temperature sensitive male sterile alleles, which precludes the recovery of nonsense mutations. For both organisms, now that the mutant phenotypes and gene structures are known, the function of C2 and other ferlin domains can be systematically tested. For instance, the effects of targeted disruptions of individual domains on phenotypes or the ability of individual isoforms to rescue mutant phenotypes can be evaluated.

Finally, we note that while ferlin genes are expressed in the gonads and required for fertility in both *D. melanogaster *and *C. elegans *[2,15], this gene family has not yet been implicated in fertility in mammals. To date, studies of mammalian ferlins have focused largely on tissues that show a disease phenotype caused by ferlin mutations and a role in the gonad has not been explored. However, ferlin transcripts have been detected in the testis and male germline of colts [35] and mice [36], and an antibody that recognizes mouse OTOF shows that this protein is located in the testis [9]. It will be interesting to determine if one or more of the mammalian ferlin proteins, like those in *D. melanogaster *and *C. elegans*, play a role in fertility. Alternatively, ferlins whose original function in animal evolution may have been specific for the gonad, may have been co-opted for entirely new uses in mammals.

## Conclusion

We found that a *D. melanogaster *ferlin gene is important for male and female fertility and carries out its functions using multiple testis and ovary promoters, alternatively spliced mRNAs, and protein isoforms. Genetic analysis of a spectrum of *mfr *mutations shows that expression in males is required for efficient breakdown of the sperm plasma membrane and completion of sperm activation during fertilization. In females, a subset of *mfr *mutations perturbs egg patterning by limiting the localization of Gurken, a key ligand in this pathway, and delays the progression of maternally controlled stages of embryogenesis. These findings provide the first evidence for diverse roles of ferlins in both male and female reproduction and help establish *D. melanogaster *as model system to study the complex regulation and function of ferlins.

## Methods

### *D. melanogaster *strains and transgenes

Strains are described on FlyBase [37] or by Wilson et al. [24]. The *mfr *mutations (*mfr*^*Z*2713^, *mfr*^*Z*0695^, *mfr*^*Z*2942^, *mfr*^*Z*1021^, *mfr*^*Z*6248^, *mfr*^*Z*1386^, *mfr*^*Z*4070^, *mfr*^*Z*3281^, *mfr*^*Z*3323^, *mfr*^*Z*1250^, and *mfr*^*Z*4901^) were recovered in a screen for ethyl methanesulfonate (EMS)-induced male-sterile mutations in a *bw*;*st *background [17] from the Zuker collection [38]. The *mfr*^1 ^stock was provided by T. Ohsako and M-T. Yamamoto [16].

The two deficiency chromosomes, *Df(3L)h-i22 *and *Df(3L)ED4415*, were used in this study. Although they behaved identically in failing to complement the male sterility associated with each *mfr *mutation, *Df(3L)h-i22 *was variable compared to *Df(3L)ED4415 *in complementation assays of *mfr/Df *females. Both chromosomes lack the *mfr *gene, as verified using a PCR assay on embryos homozygous for the deficiencies (K. Okada, personal communication). Therefore, we attributed the difference to a possible genetic suppressor of the female phenotype in the *Df(3L)h-i22 *stock and used *Df(3L)ED4415 *for assaying hemizygous female genotypes.

Transgenic lines containing the *mfr *genomic region were constructed using a 10.7 kb BssHII/BsiWI fragment isolated from BACN18D24 (Children's Hospital Oakland Research Institute, Oakland, CA). After attachment of *Not*I linkers (New England BioLabs, Ipswich, MA), the fragment was cloned into the pCasPeR4 transformation vector. Standard germ line transformation techniques and genetic crosses were used to construct two independent strains (A and B) of the genotype *w*^1118^; *P [w*^+*mc*^*mfr*^+*t*10.7^]*A *or *B/SM1; st mfr*^*Z*0695^/*TM6 *and test for rescue of the *mfr *mutant phenotypes.

### Analysis of *mfr *transcripts

RNA was harvested from testes, ovaries, and carcasses of male and female flies that were 1–3 days old using the RNeasy kit (Qiagen, Valencia, CA). PolyA+ RNA was isolated by the MicroPoly(A) Purist Kit (Ambion, Austin, TX). To determine the tissue specificity of *mfr*, first strand cDNA synthesis was performed with SuperScript II Reverse Transcriptase (Invitrogen, Carlsbad, CA). RT-PCR was carried out on cDNA with *mfr *gene specific primers (located in exon 8 and 14 in testis cDNAs, and exon 7 and 13 in ovary cDNAs) and *yip-6 *control primers that span an intron. Sequences of these and other primers used in this study are available upon request. PCR products from the testes and ovaries were cloned in the TOPO-TA vector (Invitrogen, Carlsbad, CA), and generated clones were sequenced using Big Dye Terminator Cycle Sequencing Reaction Kit (Applied Biosystems, Foster City, CA) and an ABI/PRISM 3100 Genetic Analyzer.

For RACE analysis on the testes and ovaries, first-strand cDNA synthesis was performed with PowerScript™ Reverse Transcriptase (SMART RACE cDNA Amplification Kit, Clontech, Mountain View, CA) for 5'RACE and MMLV Reverse Transcriptase (First Choice RLM-RACE Kit, Ambion, Austin, TX) for 3' RACE. For both 5' and 3' RACE, PCR products were generated with two different primer sets and cloned using the TOPO-TA vector. Generated clones were sequenced as described above. For 5' RACE, the PowerScript™ Reverse Transcriptase can add between 3–5 C residues to the first strand of cDNA, an activity that later creates ambiguity for defining the exact start site of the cDNA if the corresponding genomic sequence contains one or more Gs. This ambiguity occurred for a subset of *mfr *start sites and is noted by the brackets in Figure 1B.

Sequences determined to be at the 5' and 3' end of the *mfr *transcript by RACE analysis were used to design primer sets to isolate near full-length cDNAs. PCR was performed under conditions optimized for long products (Qiagen, Valencia, CA) with a combination of ProofStart DNA polymerase (Qiagen, Valencia, CA) and *Taq *DNA polymerase (Promega, Madison, WI), and the products were cloned into the TOPO-TA vector. Generated clones of six testis and nine ovary cDNAs were selected and sequenced as described above. Sequences of cDNAs are available in GenBank as: EF120975 (T1), EF120976 (T2), EF120977 (T3), EF120978 (T4), EF120979 (O1), EF120980 (O2), and EF120981 (O3).

### Localization of *mfr *mutations

To determine the location of the *mfr *mutations, genomic DNA was extracted from males of the *bw;st *parent strain or *mfr *strains. Overlapping primer sets were used to amplify the entire *mfr *ORF and splice junctions, except 48 bp of the 5' end of exon 2. PCR products were then sequenced. Each mutation was verified by sequencing two independent products.

### Assays for male fertility and fertilization

To assess fertility, single males were mated to three *Canton-S *(*CS*) virgin females and cultures were inspected for the presence of progeny over the course of 15 days. At least 10 males were assayed for each *mfr *allele in hemizygous combinations with *Df(3L)h-i22 *or *Df (3L)ED4415 *and for a subset of *mfr *alleles in heteroallelic combination (*mfr*^*Z*0695^/*mfr*^*Z*1386^, *mfr*^*Z*2942^/*mfr*^*Z*3281^, *mfr*^*Z*2942^/*mfr*^*Z*4070^, *mfr*^*Z*2942^/*mfr*^*Z*1386^, *mfr*^*Z*2942^/*mfr*^*Z*6248^, *mfr*^*Z*2942^/*mfr*^*Z*1021^, *mfr*^*Z*2942^/*mfr*^*Z*1250^, *mfr*^*Z*2942^/*mfr*^*Z*4901^). Control crosses used males from the *bw;st *parent strain or sibling *mfr*/*Balancer *males.

In order to compare efficiencies of fertilization of *mfr *and normal sperm, a transgene expressing the *don juan-*GFP (*dj-GFP*) sperm tail marker [39] was introduced into the *mfr *mutant background to create *w; dj-*GFP; *st mfr*/*Df(3L)h-i22 *males for five alleles: *mfr*^*Z*0695^, *mfr*^*Z*6248^, *mfr*^*Z*2942^, *mfr*^*Z*3281^, and *mfr*^*Z*2713^. Males were mated to *CS *females and laid eggs were collected up to 90 minutes after egg deposition then processed as described below to assay GFP. *dj-GFP; mfr*^+ ^males were used in a control cross to monitor the efficiency of the assay. In this cross, 98% of the eggs hatch (n = 335 eggs), and we detected the DJ-GFP sperm tail marker in 87% of the eggs (n = 60 eggs).

Eggs laid by females were dechorionated in 50% bleach, fixed in 4% paraformaldehyde with an octane overlay, devitellinized with methanol, stained with 1 μg/ml DAPI, and then mounted on slides in Vectashield (Vector Laboratories Inc., Burlingame, CA). Preparations were viewed using a BioRad Radiance 2000 LSCM confocal microscope in conjunction with a Spectra-Physics Mai Tai Laser, a 488 nm Kr/Ar laser, and a Nikon Eclipse E600-FN fluorescent scope. Z-series stacks were compiled using NIH Image and images were edited using Adobe Photoshop 7.0.

Two additional transgenes, expressing CD2 or Snky-GFP, were introduced into a *mfr*^*Z*0695 ^mutant background. Assays for protein expression in sperm were performed as described by Wilson et al. [24] except a 1:25 dilution of the anti-rat CD2 monoclonal antibody (Harlan Sera-Labs Ltd, Loughborough, UK) and a 1:200 dilution of the Alexa 488 rabbit anti-mouse and goat anti-rabbit antibodies (Alexa 488 Signal-Amplification kit, Molecular Probes, Eugene, OR) were used.

### Assays for *mfr *effects during oogenesis and embryogenesis

To assay for *mfr *effects in females, crosses were set up to generate siblings with control and *mfr *mutant genotypes. These crosses allowed morphological comparisons of eggs laid by *bw/+; st mfr*^+^/*Df(3L)ED4415 *control females to *bw/+; st mfr/Df(3L)ED4415 *females for each of the 11 *mfr *alleles, and to eggs laid by heteroallelic *bw/+; st mfr*^*Z*0695^/*st mfr*^*Z*1386^, *bw/+*; *st mfr*^*Z*0695^/*st mfr*^*Z*4070 ^and *bw/+; st mfr*^*Z*1386^/*st mfr*^*Z*4070 ^females. A minimum of 176 eggs was examined for each female genotype. Eggs were classified as having either wild-type or mutant morphology based on length and distance of the two dorsal appendages. Eggs were photographed using a Nikon photomicroscope equipped with a CoolSNAP cool-charged coupled device camera (RS Photometrics, Tucson, AZ).

Additional assays in females focused on mainly on the *mfr*^*Z*4070 ^and *mfr*^*Z*1386 ^mutations. To examine localization of Gurken during oogenesis, ovaries from *bw;st *and *bw;st mfr*^*Z*4070^/*st mfr*^*Z*1386 ^females were dissected and fixed in 4% paraformaldehyde in 1 × PBS and heptane. The ovaries were incubated overnight with a 1:100 dilution of a primary mouse Gurken antibody [40] (Developmental Studies Hybridoma Bank, Iowa City, IA) and then subsequently with a 1:1000 dilution of the Alexa 488 goat anti-mouse secondary antibody (Molecular Probes, Eugene, OR). The ovaries were stained with 0.1 mg/ml DAPI, cleared in 80% glycerol overnight at 4°C, then mounted on coded slides so scoring could be done without knowledge of the genotype. Localization of the Gurken signal was scored as abnormal if the signal did not extend posteriorly beyond the oocyte nucleus in stage 10 egg chambers.

To assess female fertility and maternal effects on embryogenesis, *bw;st, bw/+;st mfr*^*Z*1386^/*st mfr*^*Z*4070^, and *bw/+;st mfr*^*Z*4070 ^or *st mfr*^*Z*1386^/*TM6 *females were mated to wild-type *CS *males. Eggs were collected then held at 25°C for 50 min, 90 min, or 48 hours. After 50 or 90 minutes, embryos were dechorionated in 50% bleach, rinsed in 0.2% NaCl/0.2% Triton-X and water, and treated with 0.5 M EGTA. The embryos were vortexed in a 50:50 mixture of octane and methanol. The octane layer was removed and the embryos were rinsed in methanol and a step-wise increase in 0.1% Triton X in 1 × PBS. The embryos were stained in 0.1 μg/ml DAPI, placed in 90% glycerol at 4°C overnight, and staged according to the number of nuclei in the embryo. Total nuclei were counted for embryos in cycle 1 through cycle 8. After cycle 8, the total number of nuclei was extrapolated from the number of nuclei along the embryo periphery. For the embryos held for 48 hours, the hatch rate was determined by counting the unhatched embryos.

### Statistical analysis

All statistical analyses were performed with the program JMP (SAS Institute, Cary, NC). χ^2 ^likelihood ratio tests were used to compare the goodness-of-fit between models. Because multiple tests can increase type 1 error, when three pairwise comparisons were made, the sequential Bonferroni correction was used to determine the critical *P*-value (lowest *P *< 0.0167, middle *P *< 0.025, and highest *P *< 0.05). An ordinal logistic regression model was used to analyze the distribution of embryonic mitotic stages for Figure 10.

### Protein prediction programs

The predicted Mfr protein structure was evaluated by SMART [41,42]. The C2 domain closest to the C terminus (C2F) was not predicted by SMART, so the boundaries of this domain were determined by homology to predicted C2F domains of Mfr orthologs in other *Drosophila *species. Percent identity between sequences was determined with ClustalW [43].

## List of abbreviations

RACE for rapid amplification of cDNA ends

RT-PCR for reverse transcriptase-polymerase chain reaction

bp for nucleotide base pairs

kb for kilo base pairs

nt for nucleotide

Ca^2+ ^for calcium ion

## Authors' contributions

MS designed and carried out the studies, analyzed the data, and drafted the manuscript. BW isolated the *mfr *mutations, conceived of the study, participated in experimental design and data analyses, and helped draft the manuscript. Both authors read and approved the final manuscript.
